# Emission Trading System, Carbon Market Efficiency, and Corporate Innovations

**DOI:** 10.3390/ijerph19159683

**Published:** 2022-08-05

**Authors:** Rui Zhu, Liyu Long, Yinghua Gong

**Affiliations:** 1 Economics and Management School, Changsha University of Science and Technology, Changsha 410114, China; 2 Shanghai National Accounting Institute, Shanghai 201702, China

**Keywords:** carbon market efficiency, technological innovation, difference-in-difference model, impact mechanism

## Abstract

Taking China’s emission trading system (ETS) pilot in 2013 as a quasi-natural experiment, this paper uses the difference-in-differences (DID) models to study whether the regional pilot ETS can promote technological innovation in enterprises. In addition, this paper examines the influence mechanism of the ETS innovation effect, with a focus on three key dimensions of the carbon market efficiency: market price effectiveness, market product diversity, and market order normativity. The results show that the pilot ETS has significantly promoted the technological innovation of regulated enterprises, specifically, 1.405*** for the total R&D investment, and 2.783*** for the number of patent applications. Moreover, the regional carbon price has a positive moderating effect on the innovation effect of ETS. Meanwhile, the innovation effect is more significant when the gap between the carbon price and the marginal abatement cost (MAC) of CO_2_ is smaller, when the carbon financial derivatives are more abundant, or when the local market supervision is stronger. This study provides empirical evidence for the improvement of the national unified market and provides useful policy implications for developing countries to design ETS suitable for their national conditions.

## 1. Introduction

Excessive greenhouse gases emissions have massively compromised the sustainability of the natural world and economy (Ionescu, 2021; Davidson et al., 2021) [1,2], faced with multiple challenges such as the climate crisis and environmental pollution, countries around the world are actively exploring energy conservation and emission reduction policies (Zheng et al., 2020; Qian et al., 2021) [3,4], and the transition to an environmentally friendly society necessitates the establishment of low-carbon policies that meets the economic development in line with sustainable goals (Ionescu, 2021) [1]. China has become the world’s largest energy consumer and carbon emitter (IEA, 2014) [5], with plans to reach a carbon peak by 2030 and be carbon neutral by 2060. Hence, China is facing the dual challenge of effectively controlling carbon emissions while maintaining economic growth. As the largest developing country, China attempts to achieve cost-effective emission mitigation through carbon emissions trading (ETS), guiding enterprises to transform technological innovation, the progressive innovation investments can contribute to the excess economic returns of an enterprise (Ionescu, 2021) [6], and achieve high-quality economic development (Cui et al., 2021) [7], which can shed light on other emerging economies like Africa that are developing national innovation strategies and are assuming a steady increase R&D expenditures to achieve the global goals of green development (Dobrzanski et al., 2021) [8].

The Porter Hypothesis argues that strict and reasonable environmental regulations can motivate firm technological innovation (Porter and Linde, 1995) [9]. As a market-based environmental regulation, ETS encourages firms to achieve cleaner production through technological investment, radically reduce pollution emissions (Caparrós et al., 2013) [10], and create productivity benefits to offset the cost of environmental management and realize the Porter Hypothesis. Thus, in the long run, R&D investment can more effectively solve the environmental pollution problems of enterprises and achieve a win–win situation for both economy and the environment (Mitchell and Connor, 2004) [11]. Extensive studies have explored the ability of pilot ETS in China to significantly reduce regional CO_2_ emission intensity (Zhou, 2019; Xuan, 2020) [12,13], and have also confirmed the positive correlation between pilot ETS and enterprise technological innovation (Cui et al., 2021; Zhu et al., 2019) [7,14]. However, the carbon market efficiency of China’s pilot ETS is low and varies greatly across regions (Zhao et al., 2016) [15], which may greatly constrain the design and innovation effects of a national unified ETS. Without proper carbon market monitoring, the level of competitiveness within regions might decrease in the long run (Gruzauskas et al., 2020) [16]. Therefore, it is necessary to systematically evaluate the efficiency of the carbon market and conduct in-depth discussions as an important influencing mechanism for the innovation effect of ETS.

Liquidity, carbon price volatility, volume volatility, and system designs are the main aspects to discuss the market efficiency of the carbon market (Kalaitzoglou and Ibrahim, 2015; Sanin and Violante, 2015) [17,18]. China’s pilot ETS covers the eastern, central, and western regions where differences in economic endowments exist, and the pilot carbon market shows significant heterogeneity. With differences in market design, emission thresholds, and carbon allowance allocation methods in each pilot region (Yao et al., 2021) [19], resulting in obvious differences in carbon allowance price, volume, and market activity in the seven carbon markets. Considering the basic characteristics and regulations of corporate innovation behavior (Durana et al., 2020) [20], this paper focuses on three key carbon market efficiency dimensions, including carbon price, carbon market financial derivatives, and market regulation mechanisms, then explores the influence mechanism of ETS and the enterprise innovation effect.

First, the carbon price is the core of the carbon trading mechanism and has a significant impact on the overall system strategy (Ionescu, 2021) [1]. A reasonable and effective carbon price has a guiding effect on the enterprises’ technological innovation. If carbon trading cannot form a fair and appropriate price, the effectiveness of the carbon market will be greatly weakened. In addition, the carbon price is the embodiment of the social marginal abatement cost, when the carbon price is equal to the carbon marginal abatement cost (MAC), the supply and demand sides of the market can achieve complete competition. As a whole, all enterprises can achieve the emission mitigation targets at a lower cost. However, the current carbon price in China’s pilot ETS is at a low level, deviating from the carbon MAC (Ji et al., 2018) [21]. It is necessary to deeply explore how the carbon price level of each regional market will affect the innovation effect of pilot ETS, as well as the deviation between the carbon price and carbon marginal abatement cost (price-MAC-gap). Second, since carbon trading has strong financial attributes, financial institutions can indirectly participate in the carbon trading market through financial product innovations. By enriching the trading of financial derivatives, enterprises can perform better risk management. Hence, diversified market participants help optimize the efficiency of a carbon market. Carbon financial derivatives such as futures can serve as catalysts for price discovery, and by hedging against the uncertainty of future spot prices, they decrease the volatility in carbon price and can help better achieve the effectiveness of ETS (Xu et al., 2014) [22]. In addition, the carbon market is a market created by legal policies, law enforcement is the basis for the establishment and operation of the carbon market. Therefore, the design of the system will affect the efficiency of the market (Zhao et al., 2016) [15]. The local governments have defined the purpose of transactions, market participants, trading rules, and cap setting through laws and regulations, laying the legal foundation for the ETS. Therefore, the discussion of the normative nature of market regulation mechanisms can provide experience for improving the market rules of ETS.

To sum up, this paper systematically evaluates the carbon market efficiency from the three dimensions: market price effectiveness, market product diversity, and market order normativeness, and further explore the impact mechanism of ETS and corporate innovation. There are two contributions to this paper.

(1) Few studies have explored the impact mechanism of ETS and corporate innovation. This paper takes carbon market efficiency as an important impact mechanism and uses regional carbon market differences to systematically test its role. Empirical evidence is provided for the design and improvement of carbon trading markets in emerging economies.
(2) Based on the perspective of the operational effectiveness of the carbon market, this paper deeply describes the impact of the internal design of the carbon market on the Porter Hypothesis, deepens the understanding of the operational effectiveness of the carbon market and the function of resource allocation, and enriches related literature.

The remainder of this article is as follows. Section 2 describes the background of pilot ETS, mechanism analysis of its influence on firm innovation, and research hypotheses. Section 3 presents the design methodology, variables, and models. Section 4 describes the baseline results, influence mechanism regression results, and robustness tests. Section 5 outlines the conclusions and policy implications.

## 2. Background of Pilot ETS and Research Hypothesis

### 2.1. Background of Pilot ETS

**Figure 1:**
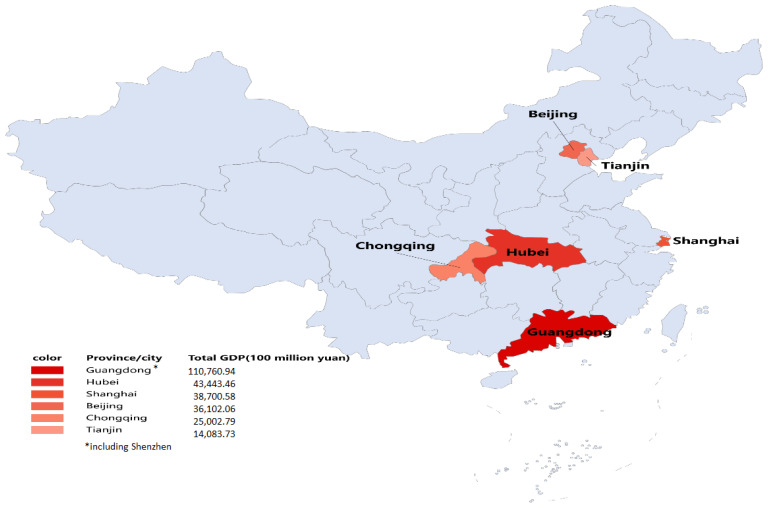
Spatial distribution of all seven pilot cities for ETS in China.

In October 2011, the National Development and Reform Commission (NDRC) carried out carbon trading pilot work in seven provinces and cities: Beijing, Shanghai, Tianjin, Chongqing, Hubei, Guangdong, and Shenzhen, as shown in Figure 1. Among them, the Shenzhen carbon market was first launched in June 2013, followed by other markets, and in the first half of 2014, all seven carbon markets were launched. By the end of 2020, the carbon markets had successfully conducted 5–6 years of compliance. In 2017, China launched a national ETS, which first covered the power sector, and in July 2021, based on the positive experiences achieved in the pilot ETS, the national carbon market officially started trading. The differences among carbon markets provide an objective foundation for us to examine the influence mechanism of China’s pilot ETS and enterprise technological innovation.

**Figure 2:**
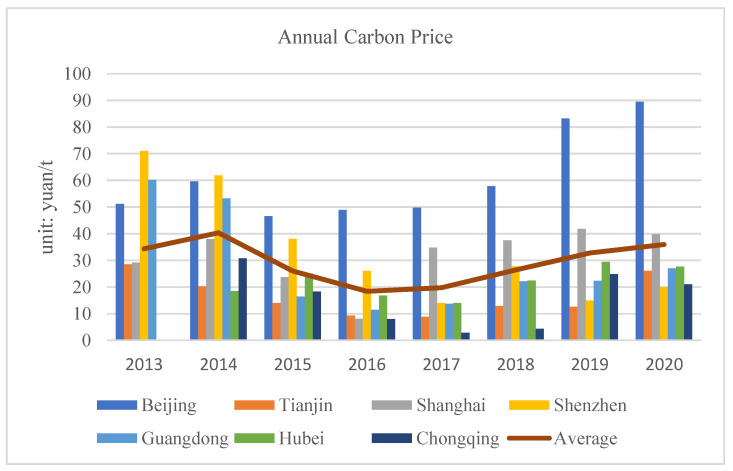
The average annual carbon price for all seven carbon pilots.

As Figure 2 shows, overall, carbon prices have been at a low level and fluctuating sharply since the trial run of China’s pilot ETS (Ji et al., 2021) [23]. During the period 2013–2020, the average trading price of the seven carbon pilots was 35.9 yuan/ton, which is not an optimal situation for China’s carbon market compared to the European carbon price, which has exceeded 50 euros/ton.

**Figure 3:**
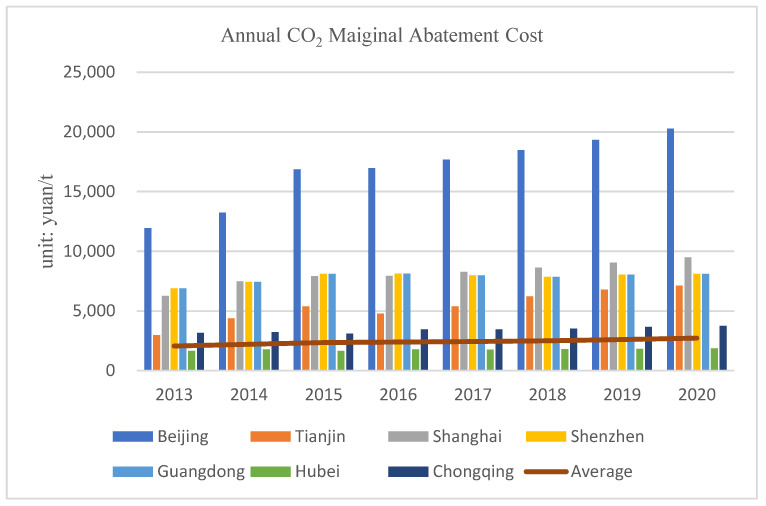
The average annual MAC across all seven carbon pilots.

Among each pilot region with different geographical locations, there is significant spatial heterogeneity at cultural, economic, geographic, and technological levels (Hu et al., 2020) [24]. In addition, as a result of diverse ecological needs and different implementation of laws and regulations, the MAC of CO_2_ shows significant regional variations (Yang et al., 2017) [25]. Referring to Wang et al., (2020) [26], Figure 3 shows the MAC of CO_2_ in each pilot provinces and cities, and it can be seen that the MAC of CO_2_ varies greatly among regions, with Beijing having the highest MAC.

**Figure 4:**
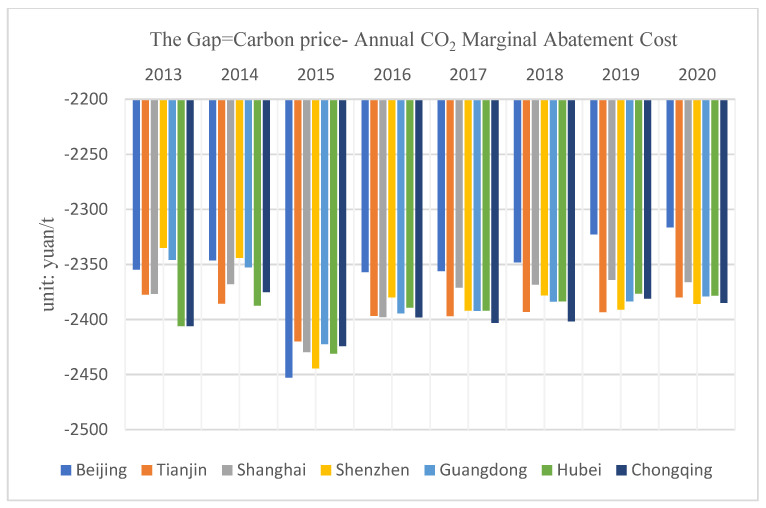
The gap between the average annual carbon price and the average annual MAC of CO_2_.

Marginal cost theory proposes that, when the carbon market optimal price is equal to the carbon MAC, the carbon price can provide an accurate market price signal for enterprises. Figure 4 shows the gap between the average annual carbon price in the pilot areas and the average annual MAC of CO_2_ (This paper only shows the gap between the average annual carbon price of all the seven pilot carbon markets and the average annual MAC of CO_2_), and it can be seen that there is a large deviation between the carbon price of each pilot area and the average annual carbon MAC, and the carbon prices of various provinces and cities are at a low level, with much room for improvement.

### 2.2. Research Hypothesis

The concept of ETS originated with the economist Dales in the 1990s. Dales (1968) [27] introduced the theory of property rights based on Coase’s theorem into environmental pollution control, arguing that pollution is a property right granted by the government to enterprises, and pollution rights can be transferred to enterprises through market means, forcing enterprises to carry out environmental innovation. Before the implementation of ETS, high carbon-emitting enterprises faced relatively lenient carbon emission reduction constraints, while the introduction of market measures to control CO_2_ emissions, increased the actual carbon emission costs, production costs, and expected future costs for enterprises. As a market-based environmental mechanism, ETS achieves radical mitigation by internalizing the external costs of corporate environmental pollution and incentivizing enterprises to innovate in green technology through the “innovation compensation effect”. Environmental green innovation represents an essential driver of sustainable development of companies, and when corporations face severe financing constraints, the performance of green technological innovation can be compromised (Ionescu, 2021) [28]. While ETS provides sustainable, dynamic economic incentives for corporate technological innovation and promotes investment in emission-reducing technologies. From the international perspective, especially if accompanied by the use of environmental technologies, the CO_2_ emissions will be reduced (Davidson et al., 2021) [2]. Therefore, as a profit-seeking enterprise, funds are increasingly invested in green and environmental innovation (Ionescu, 2021) [28], and it will improve its resource allocation efficiency through technological innovation (Bu et al., 2020) [29]. Based on the perspective of dynamic incentives, ETS, as a market-based environmental regulatory policy method, can promote technological research and development and innovation of enterprises compared with mandatory policies. On this basis, Hypothesis 1 is proposed.

**Hypothesis** **1** **(H1).** *The implementation of the ETS has a positive effect on the technological innovation of enterprises.*

Market-based environmental regulatory policies need to rely on well-functioning market mechanisms to function better (Kathuria, 2006) [30], and exploring the efficiency of the market in seven carbon markets is a powerful illustration of the innovative effects of ETS (Zhang et al., 2020) [31]. Based on the effectiveness of the market price, in the ETS, the carbon allowance price is the crucial factor in determining whether the market mechanism is perfect and whether regional emission reduction targets can be achieved (Tang et al., 2019) [32]. Due to the poor carbon price, it is difficult to effectively promote emission reduction technological innovation in regulated enterprises; too high a carbon price will put too much pressure on enterprises to reduce emissions and affect their output. Too much volatility in carbon prices increases market risks and makes it difficult to provide long-term emission reduction signals for enterprises, and the effectiveness of market prices can effectively inhibit corporate carbon emissions and promote enterprises to invest in green and low-carbon industries.

Nevertheless, the practical experience of the world’s major ETS shows that the deviation of carbon prices from policy expectations is one of the most prominent problems in the operation of the ETS. At this stage, China’s carbon price is at a low level and varies from region to region, which will have an impact on the effective implementation of the ETS. In the pilot areas where the carbon price is relatively high, the higher carbon prices will increase the transaction costs of enterprises, which will be transferred to the production and manufacturing costs of products and erode the profitability of products, leading to a decline in the business performance of enterprises, at this time, the climb in the carbon price will have a positive moderating effect on the technological innovation because the process improvement brought by technological innovation will save costs and reduce carbon emissions in the long run (Ang et al., 2009) [33]. To assess the impact of market price effectiveness on the innovation effect of the ETS, this paper proposes Hypothesis 2 based on the average annual carbon price of each carbon market.

**Hypothesis** **2** **(H2).** *The higher the carbon price in the regional carbon market, the stronger the promotion of the ETS on the technological innovation of enterprises.*

In addition, according to the price theory, in a perfectly competitive market, the carbon price is determined by its marginal emission reduction costs, and the optimal carbon price is the smallest carbon MAC that can achieve the emission reduction target (Tang et al., 2019) [32]. When the equilibrium price of carbon is equal to the MAC of CO_2_, the effectiveness of market prices can enhance the liquidity of the carbon market, the market performance is better, and fully realize the successful allocation of market resources. In this case, most carbon markets have over-allocated allowances, carbon prices are still relatively thin, and carbon prices deviate from carbon MAC, which can provide a benchmark for determining the optimal carbon price for each pilot (Liu et al., 2021) [34].

**Figure 5:**
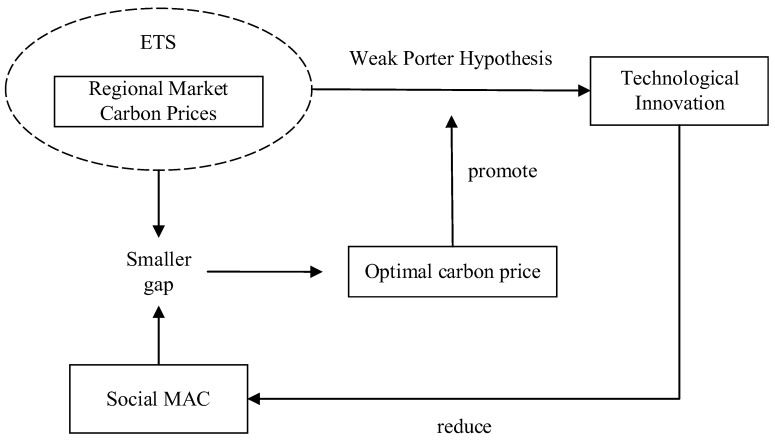
The overall theoretical framework diagram.

The main purpose of China’s implementation of ETS is to achieve the carbon emission reduction target at a minimal social cost for the local government. When the carbon market operates effectively, it can drive enterprises with low abatement costs to accelerate emission reduction, to minimize the total social abatement cost, which has the effectiveness of abatement costs. As shown in Figure 5, with the continuous improvement of the carbon market, the carbon price at a low level continues to rise, which will narrow the gap with the carbon MAC, and when the optimal carbon price level is reached, it can better enhance the enthusiasm of ETS for energy conservation and emission reduction and technological innovation of enterprises. At the same time, the behavioral choices of enterprises in the face of emission reduction constraints have a direct impact on the abatement costs (Wu et al., 2014) [35], and when enterprises significantly increase green technological innovation, making low-carbon technologies cheaper due to economies of scale, will reduce the MAC of the entire region (Kojima et al., 2021) [36], which will have a positive impact on reducing the MAC of the entire society, this virtuous circle will lead to significant emission reductions. Most of the existing literature focuses on the calculation of the MAC over a certain period or in a particular industry, and this paper proposes Hypothesis 3 based on the price-MAC-gap.

**Hypothesis** **3** **(H3).** *The smaller the price-MAC-gap, the stronger the promotion of the ETS on the technological innovation of enterprises.*

Another concern for the efficiency of the market is the diversity of market financial derivatives, and carbon financial derivatives are indispensable trading products in the carbon financial market. International experience shows that the diverse portfolio of carbon financial derivatives increases the activity of the carbon trading market (Liu et al., 2010) [37], and they can effectively avoid the risk of trading price fluctuations and increase the liquidity of the market (Zhou et al., 2019) [38]. Rittler (2012) [39] studied the relatively successful EU-ETS and argued that the futures markets led the price discovery in the spot markets, and the futures markets drive a major part of price discovery in the spot market.

For enterprises and industries, the clearer carbon price expectations provided by carbon futures can help reduce the pressure on enterprises to transform and upgrade. Enterprises can use carbon futures to manage risks, lock in carbon costs in advance, and engage in technological innovation and energy conservation, and emission reduction. Since the pilot ETS in 2014, the number of carbon financial derivatives offered by each pilot carbon market has varied significantly, and to assess the impact of market financial derivatives diversity on the innovation effect of ETS, this paper collects the number of carbon financial derivatives in each pilot region. Based on this, Hypothesis 4 of this paper study is proposed.

**Hypothesis** **4** **(H4).** *The more carbon financial derivatives in the regional carbon market, the stronger the promotion of the ETS on the technological innovation of enterprises.*

As an important governance method for the Chinese government to deal with environmental issues such as global warming, ETS requires market design and policy intervention, and the normality of market regulation mechanisms will have an important impact on the operating efficiency of the carbon market. The effective implementation of market-based environmental policies is closely related to the implementation of regional environmental law enforcement (Jacobsen et al., 2016) [40]. A strong and rigorous legal system of environmental regulation can largely avoid the short-sighted behavior of enterprises (Bénabou and Tirole, 2010) [41] and positively influence the environmental governance behavior of enterprises. The success of EU-ETS is attributed to the design of its trading system and the constraints of relevant mandatory laws and regulations (Zhou et al., 2017) [42].

Since the pilot ETS, seven localities in China have attached great importance to the construction of a legal system for carbon trading and organized relevant departments to carry out various groundwork, including the establishment of special management agencies and the formulation of local regulations (Chen et al., 2021) [43]. However, China’s seven carbon market pilots span across the eastern, central, and western regions, and the economic differences, natural environment, geographical characteristics, and environmental quality in these three regions determine the regulatory content design and local environmental supervision in each region are different. To assess the impact of market regulation mechanism normativity on the innovation effect of ETS, this paper collects the environmental regulatory policy documents related to the construction of carbon markets issued by each pilot region (including the implementation rules of carbon emission quota management and the implementation plan of the pilot work), etc., to measure the normality of environmental regulation in each pilot region. Based on this, Hypothesis 5 of the research in this paper is proposed.

**Hypothesis** **5** **(H5).** *The stronger the environmental regulation of the regional carbon market, the stronger the promotion of the ETS on the technological innovation of enterprises.*

## 3. Methodology and Data

To overcome the endogeneity problem of reverse causality of the empirical processes, this paper selects the DID method, which is widely used in the field of policy evaluation, to evaluate the impact of the pilot ETS on enterprise technological innovation. The regulated enterprises in seven provinces and cities of Beijing, Tianjin, Shanghai, Chongqing, Hubei, and Guangdong, which are included in the China pilot ETS, are regarded as the treatment group, and these enterprises are involved in the chemical, building materials, non-ferrous metals, papermaking, petrochemical, steel, electric power, and aviation industries, while other enterprises in the above eight industries in the non-pilot regions are considered as the control groups. Given that the official launch of the carbon market in the pilot regions is scheduled for the second half of 2013-the first half of 2014, this study considers 2014 and beyond as the policy implementation year.

### 3.1. Empirical Model

The baseline DID model is constructed as follows:

(1) $$lnRD_{it}/lnpatent_{it}=\alpha_{0}+\alpha_{1}treat_{i}*post_{t}+\alpha_{2}treat_{i}+\alpha_{3}post_{t}+\alpha X_{it}+\varepsilon_{it}$$

where the dependent variables are *lnRD_it_* and *lnpatent_it_*, which measures the enterprise technological innovation, indicating the total R&D investment and total number of patent applications of enterprise *i* in the time *t*, which are logarithmically treated; *treat_i_* is a dummy variable that denotes whether the firm is included in the regulated enterprises in the pilot list, *treat_i_* is 1 if the enterprise *i* is a regulated enterprise, otherwise 0; the variable *post_t_* indicates whether the policy implementation year is after 2014 or not; the value is 0 if before 2014, otherwise 1; The estimated coefficients *α*_1_ of the multiplication term treat_i_*post_t_, *α*_2_, *α*_3_ are DID estimators, indicating the net impact of the pilot ETS, coefficient α_1_ is the average impact effect of pilot ETS that this paper focuses on; *X_it_* is a series of corporate control variables, including enterprise size, profitability, leverage, enterprise age, ownership structure, company growth capacity, operating cash flow, investment opportunities, the coefficients *α* represent the effect of the *X_it_* on the enterprise technological innovation. *ε_it_* represents a random error term. In addition, to minimize the impact of the industry environment and economic cycle on the technological innovation of enterprises, this paper further controls the year’s fixed effect *γ_t_*, which is used to control the impact of time-varying factors at the macro, and the industry fixed effect *μ_i_*, which is used to control for unobservable factors at the macro that does not follow the industry-level.

To verify the Hypothesis H2 and H3 and further explore the effects of carbon market price and price-MAC-gap on the technological innovation effect of the pilot ETS, a DDD model is constructed based on the DID model, and the specific regression model is as follows:

(2) $$\begin{array}{l} lnRD_{it}/lnpatent_{it}\\ \;=\alpha_{0}+\alpha_{1}treat_{i}*post_{t}+\alpha_{2}treat*price_{it}+\alpha_{3}post*price_{it}+\alpha_{4}treat\\ \;*post*price_{it}+\alpha_{5}price_{it}+\mu_{i}+\gamma_{t}+\alpha_{6}X_{it}+\varepsilon_{it} \end{array}$$

(3) $$\begin{array}{l} \;\;lnRD_{it}/lnpatent_{it}\\ \;=\alpha_{0}+\alpha_{1}treat_{i}*post_{t}+\alpha_{2}treat*lngap_{it}+\alpha_{3}post*lngap_{it}+\alpha_{4}treat\\ \;*post*lngap_{it}+\alpha_{5}lngap_{it}+\mu_{i}+\gamma_{t}+\alpha_{6}X_{it}+\varepsilon_{it} \end{array}$$

where the *price_it_* in the model (2) is the moderating variable because the specific price of carbon trading by each enterprise is not available, this paper takes the annual average carbon price of the carbon market as the carbon trading price faced by enterprises in the pilot area *i* in *t* years, and the coefficient *α*_4_ of treat*post*price_it_ is the standard DDD estimate, which reflects the moderating effect of the average annual carbon price; In model (3), *lngap_it_* is the moderating variable, which indicates the price-MAC-gap in the pilot carbon market and is treated logarithmically. The coefficient *α*_4_ reflects the moderating effect of the price-MAC-gap. In addition, the fixed effect *γ_t_* and the industry fixed effect *μ_i_* are controlled for macro-level factors that do not change over time and industry-level unobservable factors, and the definition of other variables is the same as that of the model (1).

To verify the Hypothesis H4, the DDD model constructed in this paper is as follows:

(4) $$\begin{array}{l} lnRD_{it}/lnpatent_{it}\\ \;=\alpha_{0}+\alpha_{1}treat_{i}*post_{t}+\alpha_{2}treat*product_{it}+\alpha_{3}post*product_{it}\\ \;+\alpha_{4}treat*post*product_{it}+\alpha_{5}product_{it}+\mu_{i}+\gamma_{t}+\alpha_{6}X_{it}+\varepsilon_{it} \end{array}$$

where *product_it_* represents the number of carbon financial derivatives. Coefficient *α*_4_ is the standard DDD estimate for evaluating the impact of the number of carbon finance derivatives on the pilot ETS and corporate technological innovation, and the definition of other variables is the same as above.

To verify the Hypothesis H5, the DDD model constructed in this paper is as follows:

(5) $$\begin{array}{l} lnRD_{it}/lnpatent_{it}\\ \;=\alpha_{0}+\alpha_{1}treat_{i}*post_{t}+\alpha_{2}treat*regu_{it}+\alpha_{3}post*\;regu_{it}+\alpha_{4}treat\\ \;*post*regu_{it}+\alpha_{5}regu_{it}+\mu_{i}+\gamma_{t}+\alpha_{6}X_{it}+\varepsilon_{it} \end{array}$$

where *regu_it_* is the number of laws and regulations on the carbon market in the pilot region to which the enterprise belongs, treated in logarithms. We remain interested in coefficient *α*_4_, which estimates how the effect of the pilot ETS on enterprise technological innovation is affected by the strength of regional environmental regulations, with the remaining variables being the same as above.

### 3.2. Variables and Data Sources

The existing literature usually measures enterprise technological innovation from the perspective of input and output, and innovation input is mainly measured by the amount of R&D investment (Durana et al., 2020) [44] and the intensity of R&D investment, while innovation output mainly includes the number of patent applications or authorizations and the output of new products (Brunnermeier and Cohen, 2003) [45]. Considering the time lag between patent application and final authorization, this paper selects the total R&D investment and the total number of patent applications as the measurement indexes of enterprise technological innovation.

Since the policy time chosen for this study is 2014, this paper selects panel data from 2010–2020 as the research sample. Since the enterprises included in the pilot ETS are local high-carbon emitters, this paper manually collects a list of the regulated enterprises in seven carbon markets. The list of regulated enterprises is updated (increased or decreased) every year. Considering the availability of data, this paper selects the list of the first batch of regulated enterprises published by the Development and Reform Commission of the pilot provinces and cities, and under the usual practice, we have screened the initial sample as follows: (1) Exclude ST and ST* samples; (2) Exclude financial and insurance listed companies; (3) Exclude enterprises that have been written off in subsequent years; (4) Exclude enterprises with serious lack of indicators.

The total R&D investment of enterprises comes from the WIND database, and the total number of patent applications comes from the State Intellectual Property Office (SIPO); another firm-level characteristic variable data comes from CSMAR; indicators related to the operating efficiency of the carbon market, the average annual carbon price, the number of carbon financial derivatives and the strength of environmental regulations in the pilot regions are sorted out from the seven carbon exchanges (For non-ETS regions, the average annual carbon price is 0, the number of carbon financial derivatives is 0, and the strength of environmental regulations associated with the carbon market is 0); data at the regional level are derived from the National Bureau of Statistics. The definitions of the variables are listed in Table 1.

## 4. Results and Discussion

### 4.1. Summary Statistics Analysis

**Figure 6:**
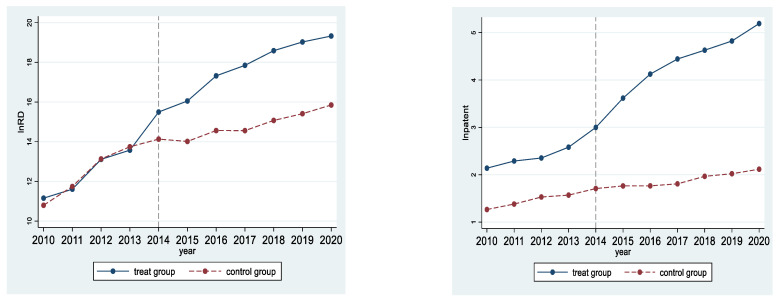
The time change trend in enterprise R&D technological innovation.

Figure 6 reflects the trend of total R&D investment and total number of patent applications of the regulated enterprises (treat group) and enterprises not included in the ETS (control group) before and after the implementation of the pilot ETS in 2014.

From Figure 6 (left), it can be seen that before 2014, the change trends of the total R&D investment of the treat group and the control group are basically the same, while after 2014, when the pilot ETS is implemented, the total R&D investment of the treat group has increased significantly, and is higher than that of the control group; in Figure 6 (right), the total number of patent applications in the treat group and the control group maintain a parallel and consistent change trend before 2014, and the total number of patent applications in the treat group has increased significantly after 2014, while the total number of patent applications in the control group has changed in a relatively flat trend.

Table 2 shows the descriptive statistics of the variables, divided into full samples and sub-samples before and after the pilot ETS. First of all, the standard deviations of the total R&D investment (lnRD) and the total number of patent applications (lnpatent) during the full sample period are relatively large, indicating that the level of technological innovation varies greatly among enterprises; the mean value of the variable treat is 0.222, indicating that 22.2% of the enterprises in the whole sample are included in the pilot ETS; the mean value of the variables post is 0.636, indicating that the sample size before and after the pilot is relatively balanced. As for lnRD and lnpatent, it can be found that before ETS, the mean value of lnRD is 12.358, and after ETS, the mean value of lnRD rises to 15.437; the mean value of lnpatent before ETS in 2014 is 1.637, and increases to 2.408 after ETS, indicating that the regulated enterprises’ technological innovation has improved significantly compared to that before the pilot ETS.

### 4.2. Baseline Regression Results

Based on the models (1) and (2) designed above, Table 3 reports the regression results of the pilot ETS on total R&D investment (lnRD) and the number of patent applications (lnpatent). First, columns (1) and (4), which no the firm-level control variables, year, and industry fixed effects, reveal that the coefficients of the treat*post are 2.858 and 1.478, respectively, and both are significant at the level of 1%; in columns (2) and (5), with the inclusion of firm-level control variables, the coefficients of treat*post are 2.822 and 1.407, respectively, which are still significant at the 1% level; in the remaining two columns, further controlling for year and industry fixed effects, the coefficients of treat*post are 2.783 and 1.405, with no change in the significance level. It is worth noting that after controlling the year fixed effect, the effect of the post is absorbed by the year effect, and its coefficient is missing due to collinearity.

### 4.3. Carbon Market Efficiency Regression Results

The implementation of ETS has significantly promoted technological innovation of enterprises, and according to H2–H5, the efficiency of the carbon market will have an impact on the ETS innovation effect. This paper selects the DDD models (2)–(5) to explore the impact of carbon market price, price-MAC-gap, the number of carbon financial derivatives, and the strength of regional environmental regulations on the innovation effect of ETS.

#### 4.3.1. The Effect of Market Price Effectiveness-Carbon Market Prices

Table 4 reports the regression results for model (2). Focusing on the coefficients of the treat*post*price, we find that the coefficients are 0.341 and 0.059 in columns (1) and (4) without controlling for firm-level control variables, year and industry fixed effects, respectively, and are significantly positive at the 1% and 5% levels, respectively; in columns (3) and (6), with the inclusion of firm-level control variables and year and industry fixed effects, the coefficients of treat*post*price are 1.585 and 0.248, both significant at the 1% level.

#### 4.3.2. The Effect of Market Price Effectiveness-Price-MAC-Gap

Table 5 reports the effect of the price-MAC-gap on ETS technological innovation. It can be found that in columns (1) and column (4), without control variables and fixed effects, the coefficients of treat*post*lngap are −0.075 and −0.141, respectively, and both are significantly negative at the levels of 1% and 5%, respectively; in columns (3) and (6), after controlling the firm-level control variables and the fixed effects of the year and industry, the coefficients of the treat*post*lngap are −0.307 and −0.591, respectively, and both are significantly negative at the 1% level.

#### 4.3.3. The Effect of Market Financial Derivatives Diversity

Table 6 reports the effect of carbon finance derivatives on the ETS innovation effect. It can be found that when there are no control variables, year and industry fixed effects in columns (1) and (4), the coefficients of treat*post*product are 0.405 and 1.234, respectively, both of which are significantly positive at the 1% level, and after controlling for the relevant control variables and fixed effects, the coefficients of treat*post*product are 0.155 and 1.008, respectively, and are still significantly positive at the 1% level, indicating that with the implementation of the pilot ETS, the regulated enterprises in pilot regions with more trading varieties increased the total amount of R&D investment and the total number of patent applications.

#### 4.3.4. The Effect of Market Regulation Mechanisms Normativity

Table 7 reports the effect of regional environmental regulation mechanisms normativity on the ETS innovation effect. In columns (1) and (4) of Table 7, the coefficients of treat*post*regu are significantly positive at the 5% level without controlling firm characteristic variables, year and industry fixed effects; when the firm-level control variables, industry and year fixed effects are also added, the coefficients of the treat*post*regu in columns (3) and (6) are 2.33 and 2.012, respectively, which are significantly positive at the 1% level. With the implementation of the pilot ETS, the regulated enterprises in the pilot regions with stronger environmental regulations have increased the total R&D investment and the total number of patent applications.

### 4.4. Robustness Test

To verify the robustness of the empirical results, a series of robustness tests are conducted in this section.

#### 4.4.1. Parallel Trend and Dynamic Effect Test

**Figure 7:**
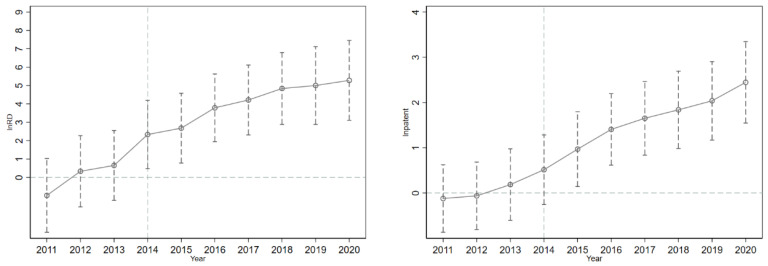
Parallel trend and dynamic effect in enterprise R&D technological innovation.

The DID method is based on the premise that the trends in the treatment group and control group must be the same before the policy is implemented, and if the model does not satisfy the assumption of parallel trend, the estimated policy effect will be biased. In Section 4.1, Figure 6 provides a preliminary demonstration that the DID design satisfies the parallel trend assumption. To further validate the validity of the DID model, and to observe how the effect of pilot ETS on enterprises’ technological innovation changes dynamically over time, we extend the model (1) to the model (6).

(6) $$lnRD_{it}/lnpatent_{it}=\beta_{0}+\sum_{t=2010}^{t=2020}\beta_{t}d_{it}+\beta_{1}treat_{i}+\beta_{2}year_{t}+\beta X_{it}+\varepsilon_{it}$$

where *d_it_* denotes treat_i_*post_t_, assuming that the pilot ETS is launched from 2010-2013, and *β*_2010_-*β*_2013_ is the corresponding policy effect. If *β*_2010_-*β*_2013_ is close to 0, indicating that the total R&D investment and the total number of patent applications in the treat and control groups do not differ significantly between 2010 and 2013, then the DID model satisfies the parallel trend assumption. In addition, the coefficients *β*_2014_-*β*_2020_ reflect the dynamic effects of ETS. In Figure 7, the *β*_2011_-*β*_2013_ coefficients are close to 0 and largely insignificant, and the parallel trend assumption is supported. The coefficient *β*_2014_-*β*_2020_ is significantly not 0.

#### 4.4.2. PSM-DID Results

To solve the potential sample self-selection bias, the robustness of the empirical results is tested by the PSM-DID method. Before the DID analysis, the samples are screened by the PSM method. First, covariate variables are selected, including characteristic factors affecting the technological innovation of enterprises, such as enterprise size, profitability, leverage, ownership, age, and factors at the regional macro level, such as regional per capita GDP level and industrial structure (regional secondary industry GDP/regional total GDP); then, the score is estimated according to the covariate variable, and the control group is matched among the enterprises in the same industry in the non-pilot regions according to the propensity score value; finally, the matching samples are re-run in DID to verify the robustness of the conclusions.

Table 8 shows the regression results of PSM-DID, in columns (1) and (4), the coefficients of treat*post are 3.067 and 1.543 without control variables, year and industry fixed effects, respectively, both of which are significantly positive at the 1% level, and in columns (3) and (6), after controlling for firm-level control variables, year and industry fixed effects, the coefficients of treat*post are 3.334 and 1.685, respectively, which are still significantly positive at the 1% level.

#### 4.4.3. Change the Measurement of Dependent Variable

In this section, the R&D investment intensity (R&D investment/total sales revenue *100%) is used to redefine the dependent variable for robustness tests. In column (1) of Table 9 without controlling for firm-level control variables, year, and industry fixed effects, the coefficient of treat*post is 0.004, which is significant at the 10% level; in column (3), after adding firm-level control variables and controlling for year and industry fixed effects, the coefficient is 0.005, which is significantly positive at the 5% level.

### 4.5. Discussion

The baseline results of the econometric estimation confirm the Porter Hypothesis. This finding is in line with the literature, where this hypothesis is proven (Xuan et al., 2020; Zhu et al., 2019). In further analysis, we project three dimensions of carbon market efficiency, such as market price effectiveness, market product diversity, and market order normativeness. Our finding on the impact of market price effectiveness on the innovation effect of the ETS is consistent with the literature (Cui et al.,2021), that is, the carbon price has a positive effect on the ETS technological innovation. Moreover, we have revealed the price-MAC-gap impact on the innovation of the ETS, consistent with the discussion in Section 2.2 that the smaller the price-MAC-gap, the more significant the effect of the pilot ETS on the total R&D investment, and the total number of patent applications. In addition, the result that the number of financial derivatives enhances the effect of pilot ETS in promoting technological innovation is also in line with the theoretical analysis above. Last, the result demonstrates that the strength of environmental regulations enhances the effect of ETS in promoting technological innovation, which confirms Hypothesis H5, and is also consistent with the fact that the success of the EU-ETS relies on the design of its trading system and constraints of the relevant mandatory regulations (Zhou et al., 2017).

The robustness tests are also provided, first, Figure 6 profoundly indicates that the DID method adopted in this paper is desirable and similar to the DID series literature (Xuan et al., 2020; Zhou et al., 2019). The different trends between the treatment group and control group indicate that the pilot ETS has a positive impact on the total R&D investment and the total number of patent applications of enterprises. After the dynamic effect test, the result indicates that the pilot ETS has a significant contribution to promoting the total R&D investment and the total number of patent applications, and this positive effect has a cumulative dynamic effect. Referring to the literature approach (Cui et al., 2021), the PSM-DID method is used to assess the robustness of the results, verifying that after PSM-DID, the pilot ETS still promotes technological innovation. Furthermore, the reliability of the results of this paper is also demonstrated by replacing the dependent variable.

## 5. Conclusions and Policy Implications

Taking China’s pilot carbon emission trading as a quasi-natural experiment, we discuss the impact of ETS on enterprise innovation based on DID and DDD methods. In addition, the mechanism of the ETS technological innovation effect is discussed in terms of carbon market efficiency. The main conclusions are as follows:

First, the implementation of ETS has a significant contribution to promoting the technological innovation of enterprises. The results verify that market-based environmental regulation can significantly promote the total R&D investment (estimated coefficient 2.783***) and the number of patent applications (1.405***) of enterprises, and this positive effect still holds in robustness tests.

Secondly, the empirical results show that: (1) The higher the carbon price in the regional carbon market, the stronger the promotion of the ETS on the technological innovation of enterprises (1.585***, 0.248***). (2) The smaller the price-MAC-gap, the stronger the promotion of the ETS on the technological innovation of enterprises (−0.307***, −0.591***). (3) When the number of carbon financial derivatives in the carbon market is greater, the promotion effect of the ETS on enterprises’ technological innovation is enhanced (0.155***, 1.008***). (4) The stronger the environmental regulation of the regional carbon market, the stronger the promotion of the ETS on the technological innovation of enterprises (2.330***, 2.012***).

Based on the above findings, the following policy implications are further proposed:

First, according to the research in this paper, local governments can form a relatively stable and reasonable carbon price by strengthening the management and constraints of the carbon trading market, which can play a favorable role in guiding the long-term investment and technological innovation of enterprises.

Second, under the goal of achieving carbon peaking by 2030, local governments can carry out emission reduction actions according to the MAC in each region, formulate a scientific and reasonable provincial carbon emission allocation plan based on fully considering regional differences, fundamentally reduce the MAC in the region by encouraging enterprises to actively carry out technological innovation, narrow the gap between the carbon price and carbon MAC, and form a virtuous circle of both environmental and economic dividends.

Third, considering that China does not yet have a complete financial system to support the development of carbon financial derivatives, financial institutions and non-financial institutions need to jointly innovate carbon financial products, participate in and provide more comprehensive financial products and services, enrich the derivatives of the carbon financial market, and improve the carbon trading market.

Finally, the healthy and normatively operation of carbon emissions trading is inseparable from legal legislation. In the future, China’s ETS policy system should establish and improve the legal system of carbon emissions trading in line with China’s national conditions, and explore and design a collaborative governance model combining various carbon emission regulatory mechanisms.

In this paper, there are still some limitations that can be improved in future research. We mainly focus on the overall situation of listed regulated enterprises in the seven pilots, First, we do not consider the specific circumstances of each carbon market to some extent because of available data. Meanwhile, we do not compare with international carbon markets and do not conduct a comparative in-depth study of carbon trading market mechanisms. Second, the construction of key variables can be improved if data is available in the future. Specifically, instead of using the number of carbon financial derivatives and the number of environmental regulations, more detailed indicators can be produced to measure the diversity of products or the normativity of market regulation mechanisms. Moreover, future studies can explore the long-term environmental efficiency. Given the possible time lag in the response of technological innovation to policy, further research can examine the lagged effect on promoting green innovation. At the same time, future studies can combine pilot ETS with other environmental regulatory policies to explore whether the joint effects have an “incentive effect” or “crowding-out effect” on enterprises’ green innovation.

## Figures and Tables

**Table 1:** Definition and description of main variables.

Types	Variable	Description
Dependent variable	lnRD	Total R&D investment (in log)
	lnpatent	Total number of patent applications (in log)
Independent variable	treat	The value is 1 if the firm is on the ETS list, otherwise 0
	post	If after 2014, the value is 1, otherwise 0
Control variable	Size	Total assets of the firm (in log)
	ROA	Net profit after tax/Total assets
	Lev	Total liability/Total assets
	Age	Firm listing years
	SOE	If state-owned enterprises, the value is 1, otherwise 0
	Grow	(Current operating income-Prior period income)/Prior period income
	Ocf	Net cash flow from operations/total assets
	Oppor	Tobin Q
Moderating variable	price	The annual average carbon price in the carbon market
	lngap	ln(|price-MAC-gap|), the annual average carbon price in each carbon market minus the carbon annual marginal abatement cost in regions (in log)
	product	Number of carbon finance derivatives in the carbon market
	regu	Number of environmental regulatory policy documents in the carbon market (in log)

**Table 2:** Descriptive Statistics.

**Panel A: Full Samples**						
Variables	N	Mean		Sd	Min	Max
lnRD	3564	14.310		7.257	0	31.630
lnpatent	3564	2.128		2.666	0	15.170
treat	3564	0.222		0.416	0	1
post	3564	0.636		0.481	0	1
Size	3564	22.520		1.364	16.700	26.600
ROA	3564	0.028		0.100	−2.555	2.163
Lev	3564	0.501		0.253	0.012	4.395
Age	3564	13.030		6.141	0	28
SOE	3564	0.590		0.492	0	1
Grow	3564	0.079		0.276	−1	0.996
Ocf	3564	0.050		0.174	−0.576	2.950
Oppor	3564	1.854		2.192	0	69.66
price	3564	5.692		15.750	0	89.490
lngap	3564	7.784		0.007	7.748	7.786
product	3564	0.857		2.367	0	11
regu	3564	0.803		1.570	0	4.875
**Panel B: Before and after ETS**						
	Before ETS			After ETS		
Variables	N	Mean	Sd	N	Mean	Sd
lnRD	1296	12.358	7.567	2268	15.437	6.828
lnpatent	1296	1.637	2.382	2268	2.408	2.778
treat	1296	0.222	0.416	2268	0.220	0.416
post	1296	0	0	2268	1	0
Size	1296	22.157	1.352	2268	22.730	1.323
ROA	1296	0.031	0.106	2268	0.026	0.096
Lev	1296	0.524	0.293	2268	0.488	0.225
Age	1296	9.547	5.389	2268	15.025	5.637
SOE	1296	0.597	0.491	2268	0.586	0.493
Grow	1296	0.089	0.280	2268	0.073	0.274
Ocf	1296	0.041	0.170	2268	0.056	0.176
Oppor	1296	1.846	1.523	2268	1.860	2.500

**Table 3:** Impact of the pilot ETS on enterprises’ technology innovation.

	(1)	(2)	(3)	(4)	(5)	(6)
	lnRD	lnRD	lnRD	lnpatent	lnpatent	lnpatent
treat*post	2.858 ***	2.822 ***	2.783 ***	1.478 ***	1.407 ***	1.405 ***
	(0.586)	(0.569)	(0.505)	(0.246)	(0.235)	(0.196)
treat	0.007	−0.412	0.712	0.904 ***	0.759 ***	0.630 ***
	(0.4902)	(0.464)	(0.438)	(0.188)	(0.179)	(0.159)
post	2.445 ***	3.395 ***	—	0.442 ***	0.704 ***	—
	(0.292)	(0.318)	—	(0.088)	(0.098)	—
Size		0.620 ***	0.470 ***		0.370 ***	0.285 ***
		(0.108)	(0.106)		(0.057)	(0.043)
ROA		−0.541	−1.102		1.201	1.137
		(1.351)	(1.433)		(0.831)	(0.772)
Lev		−1.002	0.142		−0.103	0.245
		(0.695)	(0.657)		(0.217)	(0.196)
Age		−0.241 ***	−0.281 ***		−0.080 ***	−0.076 ***
		(0.024)	(0.026)		(0.009)	(0.009)
SOE		−0.899 ***	−0.275		−0.055	0.072
		(0.270)	(0.270)		(0.105)	(0.092)
Grow		−0.080	−0.003		−0.227	−0.123
		(0.451)	(0.448)		(0.1524)	(0.1508)
Ocf		−1.830 **	−1.884 **		−0.328 *	−0.233
		(0.829)	(0.824)		(0.169)	(0.142)
Oppor		0.049 *	0.062 **		0.046 **	0.045 **
		(0.029)	(0.030)		(0.022)	(0.020)
_cons	12.360 ***	2.073	−0.333	1.436 ***	−5.977 ***	−5.300 ***
	(0.241)	(2.184)	(2.563)	(0.067)	(1.180)	(0.881)
Ind fixed	No	No	Yes	No	No	Yes
year fixed	No	No	Yes	No	No	Yes
N	3564	3564	3564	3564	3564	3564
adj.R-sq	0.042	0.098	0.157	0.114	0.178	0.350

Standard errors in parentheses, * *p* < 0.1, ** *p* < 0.05, *** *p* < 0.01.

**Table 4:** The moderating effect of annual average carbon price in in the carbon market.

	(1)	(2)	(3)	(4)	(5)	(6)
	lnRD	lnRD	lnRD	lnpatent	lnpatent	lnpatent
treat*post*price	0.341 ***	0.520 ***	1.585 ***	0.059 **	0.097 ***	0.248 ***
	(0.057)	(0.061)	(0.114)	(0.019)	(0.020)	(0.035)
treat*post	5.410 ***	6.254 ***	12.13 ***	2.117 ***	2.295 ***	3.159 ***
	(0.726)	(0.740)	(0.886)	(0.298)	(0.276)	(0.307)
treat*price	0.329 ***	0.400 ***	0.304 ***	−0.134 ***	−0.105 **	−0.099 **
	(0.110)	(0.112)	(0.107)	(0.036)	(0.033)	(0.030)
post*price	−0.438 ***	−0.611 ***	−1.642 ***	−0.078 ***	−0.115 ***	−0.260 ***
	(0.051)	(0.056)	(0.110)	(0.015)	(0.017)	(0.034)
price	−0.240 **	−0.315 ***	−0.261 ***	0.148 ***	0.117 ***	0.102 ***
	(0.102)	(0.103)	(0.099)	(0.034)	(0.031)	(0.028)
Size		0.726 ***	0.578 ***		0.499 ***	0.377 ***
		(0.109)	(0.108)		(0.043)	(0.039)
ROA		−1.523	−1.600		1.064	1.063
		(1.480)	(1.517)		(0.889)	(0.812)
Lev		−0.667	0.575		−0.206	0.148
		(0.708)	(0.662)		(0.238)	(0.211)
Age		−0.255 ***	−0.321 ***		−0.087 ***	−0.081 ***
		(0.024)	(0.026)		(0.009)	(0.009)
SOE		−0.849 ***	−0.287		−0.083	0.0524
		(0.279)	(0.280)		(0.101)	(0.089)
Grow		−0.046	0.139		−0.220	−0.073
		(0.447)	(0.435)		(0.152)	(0.143)
Ocf		−2.442 ***	−2.436 ***		−0.348 **	−0.242 *
		(0.835)	(0.829)		(0.159)	(0.139)
Oppor		−0.042	−0.012		0.050**	0.047 **
		(0.044)	(0.03 6)		(0.023)	(0.020)
_cons	12.32 ***	−0.148	−3.276	1.436 ***	−8.708 ***	−7.124 ***
	(0.242)	(2.215)	(2.695)	(0.067)	(0.870)	(0.803)
Ind fixed	No	No	Yes	No	No	Yes
year fixed	No	No	Yes	No	No	Yes
N	3564	3564	3564	3564	3564	3564
adj.R-sq	0.047	0.099	0.153	0.114	0.192	0.355

Standard errors in parentheses, * *p* < 0.1, ** *p* < 0.05, *** *p* < 0.01.

**Table 5:** The moderating effect of the price-MAC-gap in the carbon market.

	(1)	(2)	(3)	(4)	(5)	(6)
	lnRD	lnRD	lnRD	lnpatent	lnpatent	lnpatent
treat*post*lngap	−0.075 ***	−0.116 ***	−0.307 ***	−0.141 **	−0.231 ***	−0.591 ***
	(0.130)	(0.128)	(0.141)	(0.077)	(0.073)	(0.065)
treat*post	7.620 ***	8.493 ***	2.736 ***	2.116 ***	2.293 ***	3.157 ***
	(0.588)	(0.618)	(0.797)	(0.297)	(0.276)	(0.306)
treat*lngap	−0.181	−0.341	0.254	0.319 ***	0.251 ***	0.234 **
	(0.223)	(0.211)	(0.205)	(0.085)	(0.079)	(0.071)
post*lngap	0.102 ***	0.143 ***	0.318 ***	0.019 ***	0.027 ***	0.062 ***
	(0.122)	(0.133)	(0.255)	(0.036)	(0.039)	(0.080)
*lngap*	0.080	0.248	−0.260	−0.352 ***	−0.278 ***	0.243 ***
	(0.209)	(0.198)	(0.190)	(0.079)	(0.074)	(0.066)
Size		0.624 ***	0.475 ***		0.499 ***	0.377 ***
		(0.109)	(0.107)		(0.043)	(0.040)
ROA		−0.511	−1.073		1.064	1.063
		(1.383)	(1.446)		(0.890)	(0.812)
Lev		−1.163	0.061		−0.206	0.148
		(0.719)	(0.669)		(0.238)	(0.211)
Age		−0.245 ***	−0.285 ***		−0.087 ***	−0.081 ***
		(0.024)	(0.026)		(0.009)	(0.009)
SOE		−0.726 ***	−0.181		−0.083	0.052
		(0.273)	(0.271)		(0.101)	(0.089)
Grow		−0.046	0.139		−0.220	−0.073
		(0.447)	(0.435)		(0.152)	(0.143)
Ocf		−1.875 *	−1.912 *		−0.348 *	−0.242
		(0.822)	(0.823)		(0.160)	(0.139)
Oppor		0.046	0.061 *		0.050 *	0.048 *
		(0.029)	(0.030)		(0.023)	(0.020)
_cons	−0.068	−0.193	0.202	0.274 ***	0.216 ***	0.188 ***
	(0.137)	(0.176)	(0.196)	(0.062)	(0.058)	(0.052)
Ind fixed	No	No	Yes	No	No	Yes
year fixed	No	No	Yes	No	No	Yes
N	3564	3564	3564	3564	3564	3564
adj.R-sq	0.066	0.114	0.198	0.114	0.192	0.355

Standard errors in parentheses, * *p* < 0.1, ** *p* < 0.05, *** *p* < 0.01.

**Table 6:** The moderating effect of the carbon financial derivatives in the carbon market.

	(1)	(2)	(3)	(4)	(5)	(6)
	lnRD	lnRD	lnRD	lnpatent	lnpatent	lnpatent
treat*post*product	0.405 ***	0.319 ***	0.155 ***	1.234 ***	1.061 ***	1.008 ***
	(0.050)	(0.050)	(0.052)	(0.202)	(0.187)	(0.159)
treat*post	2.461 ***	2.013 ***	0.985 ***	0.350	0.479	0.251
	(0.322)	(0.322)	(0.338)	(0.374)	(0.347)	(0.300)
treat*product	0.246 *	0.138	0.076	0.322 ***	0.286 ***	0.287 ***
	(0.127)	(0.127)	(0.130)	(0.055)	(0.051)	(0.042)
post*product	1.344 ***	1.058 ***	0.517 ***	1.406 ***	1.296 ***	1.129 ***
	(0.166)	(0.165)	(0.172)	(0.081)	(0.079)	(0.065)
product	1.042	0.833	0.318	0.423 **	0.392 ***	0.343 ***
	(1.242)	(1.261)	(1.346)	(0.025)	(0.024)	(0.020)
Size		0.841 ***	0.579 ***		0.438 ***	0.314 ***
		(0.098)	(0.091)		(0.060)	(0.047)
ROA		−3.021 *	−1.731		1.033	1.209
		(1.550)	(1.533)		(0.843)	(0.768)
Lev		−1.860 **	0.782		−0.381 *	0.161
		(0.740)	(0.651)		(0.227)	(0.199)
Age		−0.107 ***	−0.320 ***		−0.064 ***	−0.075 ***
		(0.022)	(0.026)		(0.009)	(0.009)
SOE		−1.487 ***	−0.323		−0.122	0.088
		(0.274)	(0.276)		(0.102)	(0.091)
Grow		−0.197	0.198		−0.194	−0.013
		(0.459)	(0.437)		(0.154)	(0.144)
Ocf		−2.191 **	−2.430 ***		−0.368 **	−0.288 **
		(0.871)	(0.827)		(0.165)	(0.140)
Oppor		−0.018	−0.006		0.050**	0.045 **
		(0.046)	(0.035)		(0.022)	(0.019)
_cons	13.700 ***	−1.727	−3.966 *	1.776 ***	−7.081 ***	−5.642 ***
	(0.133)	(1.992)	(2.405)	(0.043)	(1.248)	(0.970)
Ind fixed	No	No	Yes	No	No	Yes
year fixed	No	No	Yes	No	No	Yes
N	3564	3564	3564	3564	3564	3564
adj.R-sq	0.044	0.099	0.157	0.118	0.183	0.357

Standard errors in parentheses, * *p* < 0.1, ** *p* < 0.05, *** *p* < 0.01.

**Table 7:** The moderating effect of strength of environmental regulations in the carbon market.

	(1)	(2)	(3)	(4)	(5)	(6)
	lnRD	lnRD	lnRD	lnpatent	lnpatent	lnpatent
treat*post*regu	1.203 **	2.785 ***	2.33 ***	0.424 **	2.012 ***	2.012 ***
	(0.547)	(0.576)	(1.169)	(0.209)	(0.371)	(0.371)
treat*post	11.780 ***	12.360 ***	24.750 ***	8.948 ***	6.265 ***	5.724 ***
	(4.001)	(4.038)	(4.626)	(1.791)	(1.817)	(1.543)
treat*regu	15.990 ***	17.900 ***	6.609 *	7.547 ***	6.158 ***	5.646 ***
	(2.841)	(2.853)	(3.246)	(1.294)	(1.280)	(1.092)
post*regu	−6.094 ***	−7.293 ***	−14.840 ***	2.019 ***	0.340	0.407
	(1.129)	(1.147)	(1.469)	(0.496)	(0.569)	(0.489)
regu	−8.159 ***	−9.354 ***	−3.049	−3.950 ***	−3.205 ***	−2.945 ***
	(1.660)	(1.664)	(1.904)	(0.753)	(0.744)	(0.634)
Size		0.671 ***	0.599 ***		0.347 ***	0.283 ***
		(0.080)	(0.078)		(0.054)	(0.043)
ROA		−1.224	−1.783		1.339	1.294 *
		(1.416)	(1.528)		(0.832)	(0.755)
Lev		−0.443	0.686		0.099	0.237
		(0.671)	(0.644)		(0.209)	(0.194)
Age		−0.265 ***	−0.316 ***		−0.103 ***	−0.073 ***
		(0.023)	(0.026)		(0.010)	(0.010)
SOE		−0.791 ***	−0.400		0.097	0.094
		(0.269)	(0.274)		(0.106)	(0.090)
Grow		−0.161	0.133		−0.218	−0.041
		(0.459)	(0.437)		(0.153)	(0.142)
Ocf		−2.552 ***	−2.483 ***		−0.427 **	−0.279 **
		(0.832)	(0.820)		(0.174)	(0.141)
Oppor		−0.041	−0.012		0.053 **	0.043 **
		(0.042)	(0.036)		(0.023)	(0.019)
_cons	11.050 ***	0.109	1.235	0.370	−5.826 ***	−5.428 ***
	(0.517)	(1.703)	(2.229)	(0.233)	(1.189)	(0.943)
Ind fixed	No	No	Yes	No	No	Yes
year fixed	No	No	Yes	No	No	Yes
N	3564	3564	3564	3564	3564	3564
adj.R-sq	0.069	0.127	0.167	0.135	0.205	0.360

Standard errors in parentheses, * *p* < 0.1, ** *p* < 0.05, *** *p* < 0.01.

**Table 8:** The estimation results of the PSM-DID model.

	(1)	(2)	(3)	(4)	(5)	(6)
	lnRD	lnRD	lnRD	lnpatent	lnpatent	lnpatent
treat*post	3.067 ***	3.028 ***	3.334 ***	1.543 ***	1.523 ***	1.685 ***
	(0.773)	(0.754)	(0.696)	(0.246)	(0.237)	(0.211)
treat	−0.884	−1.017	0.021	0.121	0.015	−0.025
	(0.668)	(0.646)	(0.628)	(0.196)	(0.188)	(0.178)
post	1.978 ***	2.389 ***	—	0.283 ***	0.601 ***	—
	(0.584)	(0.638)	—	(0.097)	(0.138)	—
Size		0.589 ***	0.597 ***		0.383 ***	0.344 ***
		(0.132)	(0.126)		(0.081)	(0.066)
ROA		0.439	0.119		−0.457	−0.504
		(2.424)	(2.225)		(1.123)	(0.923)
Lev		−2.388 **	−0.472		−0.978 *	−0.570
		(1.061)	(0.967)		(0.516)	(0.446)
Age		−0.137 ***	−0.162 ***		−0.101 ***	−0.076 ***
		(0.038)	(0.041)		(0.014)	(0.013)
SOE		−1.097 ***	−0.345		0.043	−0.111
		(0.401)	(0.395)		(0.159)	(0.125)
Grow		−0.458	−0.518		0.115	0.237
		(0.647)	(0.601)		(0.235)	(0.222)
Ocf		−2.274	−1.560		−0.561 *	−0.298
		(1.758)	(1.441)		(0.329)	(0.266)
Oppor		0.108 ***	0.074 *		0.004	−0.004
		(0.041)	(0.039)		(0.014)	(0.015)
_cons	13.230 ***	3.068	0.710	2.228 ***	−4.830 ***	−5.101 ***
	(0.513)	(2.659)	(2.821)	(0.086)	(1.645)	(1.427)
Ind fixed	No	No	Yes	No	No	Yes
year fixed	No	No	Yes	No	No	Yes
N	1596	1596	1596	1596	1596	1596
adj.R-sq	0.073	0.112	0.294	0.104	0.180	0.422

Standard errors in parentheses, * *p* < 0.1, ** *p* < 0.05, *** *p* < 0.01.

**Table 9:** The estimation results of redefining the dependent variable.

	(1)	(2)	(3)
treat*post	0.004 *	0.005 **	0.005 **
	(0.002)	(0.002)	(0.002)
treat	0.011 ***	0.009 ***	0.012 ***
	(0.002)	(0.002)	(0.002)
post	0.005 ***	0.010 ***	0.023 ***
	(0.001)	(0.001)	(0.002)
Size		0.000	0.000
		(0.000)	(0.000)
ROA		−0.006	−0.006
		(0.004)	(0.004)
Lev		−0.013 ***	−0.010 ***
		(0.002)	(0.002)
Age		−0.001 ***	−0.001 ***
		(0.000)	(0.000)
SOE		−0.009 ***	−0.006 ***
		(0.001)	(0.001)
Grow		0.000	0.001
		(0.001)	(0.001)
Ocf		0.000	0.000
		(0.001)	(0.001)
Oppor		0.000	0.000
		(0.000)	(0.000)
_cons	0.014 ***	0.023 ***	0.024 ***
	(0.001)	(0.005)	(0.007)
Ind fixed	No	No	Yes
year fixed	No	No	Yes
N	3564	3564	3564
adj.R-sq	0.078	0.254	0.382

Standard errors in parentheses, * *p* < 0.1, ** *p* < 0.05, *** *p* < 0.01.

## Data Availability

The data presented in this study are available on request from the corresponding author.
